# Exploiting BSA to Inhibit the Fibrous Aggregation of Magnetic Nanoparticles under an Alternating Magnetic Field

**DOI:** 10.3390/ijms14035775

**Published:** 2013-03-12

**Authors:** Jian Fei Sun, Xuan Liu, Yuan Chen, Yu Zhang, Ning Gu

**Affiliations:** 1 State Key Laboratory of Bioelectronics and Jiangsu Key Laboratory of Biomaterials and Devices, School of Biological Science and Medical Engineering, Southeast University, Dingjiaqiao 87, Nanjing 210009, China; E-Mails: sunzaghi@seu.edu.cn (J.F.S.); moornorth@hotmail.com (Y.C.); zhangyu@seu.edu.cn (Y.Z.); 2 School of Medicine, Southeast University, Dingjiaqiao 87, Nanjing 210009, China; E-Mail: liuxuan@seu.edu.cn

**Keywords:** alternating magnetic field, colloidal assembly, biomolecule, bioelectronics

## Abstract

The alternating magnetic field was discovered to be capable of inducing the fibrous aggregation of magnetic nanoparticles. However, this anisotropic aggregation may be unfavorable for practical applications. Here, we reported that the adsorption of BSA (bovine serum albumin) on the surfaces of magnetic nanoparticles can effectively make the fibrous aggregation of γ-Fe_2_O_3_ nanoparticles turn into a more isotropic aggregation in the presence of the alternating magnetic field. Also, the heating curves with and without BSA adsorption under different pH conditions were measured to show the influence of the colloidal aggregation states on the collective calorific behavior of magnetic nanoparticles.

## 1. Introduction

Recently, the time-variant magnetic field was discovered to be capable of inducing the fibrous assembly of magnetic nanoparticles, even with horseradish peroxidase molecules [1–4]. This is a novel phenomenon, different essentially from the assembly induced by a magnetostatic field. However, the field-induced aggregation is not favored by some practical applications because it may interfere with the outputs. A good case in this point is the magnetic hyperthermia for the therapy of cancer [5]. Here, the magnetic nanoparticles are subject to an alternating magnetic field to generate heat which can be used as the hyperthermia. In the clinic application, before treatment, the physiatrist should establish the proper therapeutical plan on the basis of heat transfer theory to advise the clinician on the appropriate dose, time, and so on. Here, the field-induced anisotropic aggregates of magnetic nanoparticles may bring about an inaccurate calculation because the heat transfer of the anisotropic-shaped source is different from that of isotropic-shaped source. Generally, the spherical aggregates of magnetic nanoparticles are pre-hypothesized during the establishment of a therapeutical plan. If the alternating magnetic field caused the fibrous aggregation of magnetic nanoparticles, the calculated results will lead to inaccurate treatment. This is because the heating mechanism is the power loss resulting from the relaxation of magnetic moments of nanoparticles under the treatment of an external alternating magnetic field. Our group previously systematically studied the size dependence of specific power absorption [6]. The power loss can actually be expressed by:

(1) $$P={\left(mH\omega \tau \right)}^{2}/[2\tau kT\rho V(1+\omega^{2}\tau^{2})]$$

where *m* is the magnetic moment of nanoparticle, *H* is the field intensity of external magnetic field, ω is the frequency of external field, τ is the relaxation time of magnetic nanoparticles, *k* is the Boltzmann’s constant, *T* is the absolute temperature, ρ is the density of magnetic nanoparticles and *V* is the volume of nanoparticle. Here, τ, the magnetic relaxation time, is strongly dependent upon the anisotropic energy of magnetic materials which is obviously relative to the size, as well as the shape. In addition, the transfer of heat should obey:

(2) Q=-λ∇T

where *Q* denotes the thermal flux, λ is the coefficient of heat conductivity, and *T* means the absolute temperature. Because the thermal flux is dependent upon the gradient of temperature, the transfer of heat may well be relative to the shape of the thermal source. Thus, we think the shape of magnetic colloidal aggregates can affect both the generation and the transfer of heat. Regardless of whether the anisotropic aggregates lead to the quicker heating or slower heating, it will disturb the establishment of the therapeutical plan because the actual heating rate and the transfer rate are different from those in the standard model. This means the physiatrist should revise the calculation model, which is not favored in the current practice because the thermal transfer of anisotropic assemblies of nanoparticles is more complex so that the uncertainty in the clinical treatment—namely, the risk—is enhanced. In addition, the anisotropic aggregates may cause other unexpected results, such as thrombosis. This is the consequence of the aggregates of nanoparticles providing amounts of binding sites for proteins, which result in the components of blood (such as proteins and cells) aggregating onto the nanoparticle chains and further growing into thrombus [7,8]. In another study of our group, the chain-like assemblies of magnetic nanoparticles on a highly hydrophilic matrix were discovered to be greatly propitious to the adherence and the growth of several types of cells, which means the thrombosis is easy to occur if there are such assemblies in blood vessels.

Because the magnetic nanoparticles often enter into the organs through blood, the components of blood will greatly influence the surface property of the nanoparticles and thereby influence the aggregation. Here, a noticeable factor is the external magnetic field which often results in the magnetic nanoparticles aggregating more easily. Recently, it was found that the stability of magnetic nanoparticles was greatly enhanced when the nanoparticles were dispersed into the culture medium-containing serum [9,10]. The non-specific adsorption of proteins contained in the serum may be responsible for this phenomenon. However, whether the enhancement of stability can prevent the colloidal particles from aggregation in the presence of an alternating magnetic field has not yet been addressed. Therefore, whether the protein adsorption can inhibit the field-induced anisotropic aggregation is an issue worth studying. Because the surface adsorption of protein molecules can increase the repulsive interaction and weaken the magnetic interaction between the colloidal particles, we think the field-induced aggregation of magnetic nanoparticles may be inhibited or partly inhibited.

In this letter, the BSA (bovine serum albumin) was chosen as the model molecule to add into the assembly system to explore the inhibition effect. It should be mentioned that the serum was too complex to directly serve as the experimental object. Actually, we also studied the effect of the serum on the assembly of magnetic nanoparticles in the presence of alternating magnetic fields. The protein of maximum content in serum is albumin. BSA and the calf serum are cheap and convenient to obtain in the common laboratories, so we employed them here as the experimental objects rather than the human serum albumin (HSA) and human serum. The latter are more practical, but also expensive and difficult to attain. In the clinical application, the BSA obviously cannot be directly used as the capping molecule of magnetic nanoparticles because the heterogeneous protein will induce the immune response of patients. However, the overarching purpose of this paper is to study the inhibition effect of albumin in serum to the aggregation of magnetic nanoparticles in the presence of an alternating field, thus we believe the conclusion with BSA and calf serum are also applicable to the HSA and human serum.

## 2. Results and Discussion

**Figure 1:**
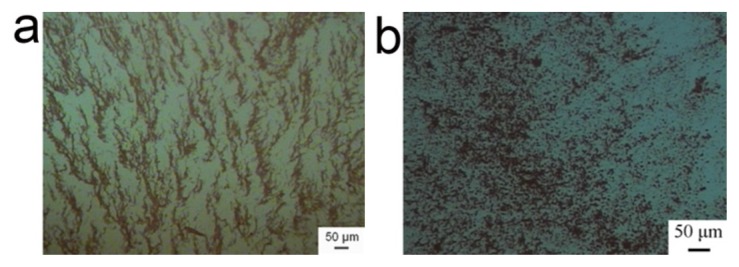
(**a**) is the optical image of γ-Fe_2_O_3_ nanoparticles aggregates without the BSA addition in the presence of a time-varied magnetic field. (**b**) is the optical image of γ-Fe_2_O_3_ nanoparticle aggregates after BSA addition in the presence of a time-varied magnetic field. The excitation current of magnetic field generator was 200 A. pH = 7 and the magnetic nanoparticles and BSA were dispersed into the pure water and their concentrations were 0.068 mg/mL and 0.02 mg/mL, respectively.

In the absence of BSA, the alternating magnetic field induced the γ-Fe_2_O_3_ nanoparticles to form the fibrous aggregates as expected (Figure 1a). However, the fibrous aggregates turned into the isotropic clusters in the presence of BSA (Figure 1b), exhibiting that the field-induced assembly was inhibited. Here, the reliability of CCD observation was guaranteed by the parallel characterization with SEM (See Supporting information, Figure S1).

**Figure 2:**
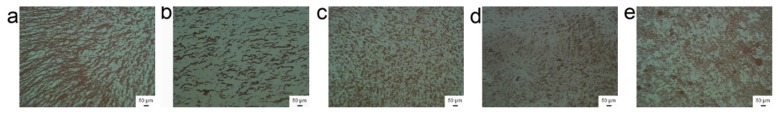
The effect of BSA volume on the field-induced assembly of γ-Fe_2_O_3_ nanoparticles. (**a**)–(**e**) samples were prepared by adding 5 μL, 10μL, 50μL, 100μL and 500 μL BSA solution into 1 mL nanoparticles suspension, respectively. The excitation current of the magnetic field generator was 200 A. pH = 7 and the magnetic nanoparticles and BSA were dispersed into the pure water and their concentrations were 0.068 mg/mL and 0.02 mg/mL, respectively.

The inhibition effect was found dependent upon the amount of BSA. When the increasing amounts of BSA were used in the experiment, there was an obvious transition from linear assembly to amorphous aggregation (Figure 2). Moreover, if the BSA molecules were added adequately, the inhibition effect was irrelative with the field strength. In our experiments, there were no obvious fibrous assemblies observed even under the highest field intensity when the adequate BSA was added (See Supporting information, Figure S2).

**Figure 3:**
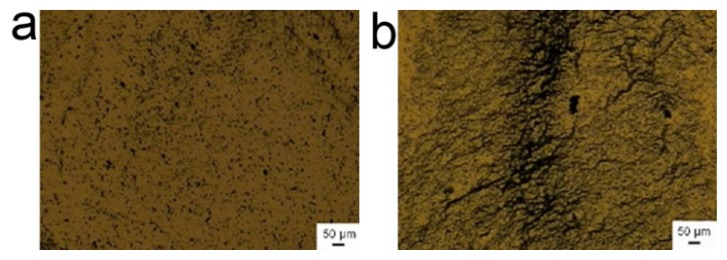
(**a**) and (**b**) are assembly images under acidic (pH = 3) and basic conditions (pH = 8), respectively. The excitation current of magnetic field generator was 200 A. The magnetic nanoparticles and BSA were dispersed into the pure water and their concentrations were 0.068 mg/mL and 0.02 mg/mL, respectively.

However, the inhibition effect of BSA molecules on the assembly of γ-Fe_2_O_3_ nanoparticles is dependent upon the colloidal pH value, even in the presence of superfluous BSA. The typical images of colloidal assemblies under acidic and basic conditions were shown in Figure 3. (The assemblies under other pH conditions were shown in Supporting Information Figure S3.) The results revealed that the inhibition effect of BSA molecules favored acidic or neutral conditions rather than the basic condition. For the basic condition, the nanoparticles still exhibited somewhat linear alignment under the treatment of the alternating magnetic field. It should be mentioned that we heresimply regarded the BSA as one chemical molecule to illustrate the mechanism of the inhibition effect. Because its property is relative to the pH value, we adjusted the pH value from 2.5 to 11 to determine the influence of pH value on the inhibition effect of BSA. In the actual applications, the pH value *in vivo* is about 7.4, and under an extremely acidic or basic condition, the BSA may be denaturalized.

**Figure 4:**
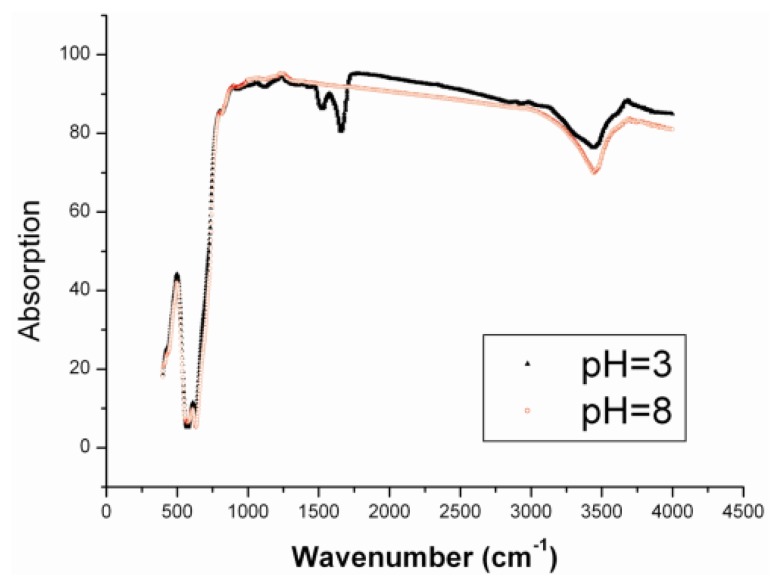
The IR spectra of nanoparticles after centrifugal separation of BSA and γ-Fe_2_O_3_ mixture under acidic and basic conditions, respectively.

It was taken for granted that this phenomenon resulted from the non-specific adsorption of BSA to the γ-Fe_2_O_3_ nanoparticles. Under the acidic condition, there was a characteristic peak of the amino group in the IR spectrum (Figure 4, 1504 cm^−^^1^), indicating that the BSA molecules absorbed on the surface of nanoparticles. However, the peak disappeared when the samples obtained under the basic condition were characterized by the IR spectrum. Here, it can be seen that the BSA can still adsorb on the surface of nanoparticles even in the cases of pH = 3 and pH = 8. Therefore, the albuminoid adsorption on the surface of nanoparticles can greatly change the field-induced assembly. We believe it results from the enhancement of repulsive interaction and weakening of attractive interaction between nanoparticles in the presence of an external field. BSA is a macromolecule. The adsorption of macromolecules augments the distance between two clusters of magnetic nanoparticles when they are magnetized by the external field, thereby preventing the two colloidal clusters from aggregating and further growing into the one-dimensional structure driven by the dipolar interaction. This experimental phenomenon also partly confirmed the assembly mechanism proposed in our previous report [1]. If the steric layer to stabilize the nanoparticles is thick enough, the alternating magnetic field is incapable of inducing the magnetic nanoparticles to form fibrous aggregates.

**Figure 5:**
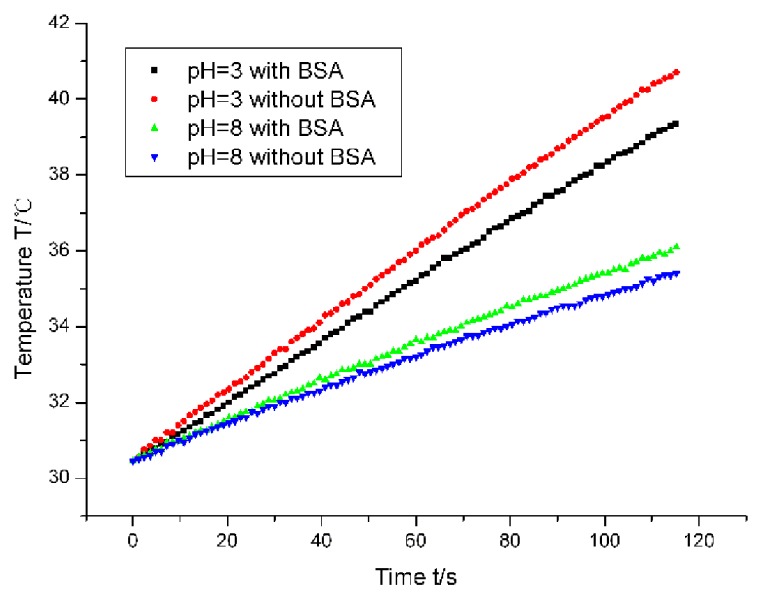
The heating curve of γ-Fe_2_O_3_ nanoparticles in the presence and absence of BSA under acidic and basic conditions. The excitation current of the magnetic field generator was 200 A.

As previously mentioned, the different aggregated states of γ-Fe_2_O_3_ nanoparticles may lead to the different behaviors in the magnetic hyperthermia. The heating curves of magnetic nanoparticles under four cases were shown in Figure 5. The time was within 2 min. Our lab previously reported that the slope of the heating curve can represent the SAR (specific absorption ratio) value, which is an indicator of the capability of nanoparticles to heat [11,12]. The colloidal samples exhibited different calorific performances, depending on the presence of BSA, as well as the colloidal pH value. Under the acidic condition, the nanoparticles with BSA adsorption had less heating power than those without BSA adsorption, indicating that the anisotropic assemblies were easier to generate heat than the spheric aggregates. However, under the basic condition, the SAR of the nanoparticles with BSA adsorption showed less difference from that of the nanoparticles without BSA adsorption. The heating power of the nanoparticles with BSA adsorption was a little higher than that of the nanoparticles without BSA adsorption. This may be correlative with the colloidal stability. Our nanoparticles were unstable in the basic condition, while the addition of BSA molecules improved the stability of the nanoparticles. It should be mentioned that the amount of magnetic nanoparticles in the heating experiments is relatively smaller than that in the practical clinic experiments. Here, the concentrations of BSA and the γ-Fe_2_O_3_ nanoparticles are chosen based on the aforementioned experiments of morphological characterization. Thus, it possibly seemed that the anisotropic aggregation induced an insignificant variation. However, based on the analysis of heating mechanism, we think the anisotropic aggregation actually contributed to the variation. The insignificant difference is due to the small amount of magnetic nanoparticles.

The thermogenesis of magnetic nanoparticles under an alternating magnetic field is due to the Neel relaxation and Brownian relaxation. Brownian relaxation is the main reason behind thermogenesis for the very small nanoparticles, meaning that the particle is motional while the magnetic moment is immovable. The magnetic moment is to reverse along with the nanoparticle. The Neel relaxation means the particle is immovable while the magnetic moment is to reverse independently. Generally speaking, the Neel relaxation brings about more heat than the Brownian relaxation. However, if the size of the magnetic materials is too large, the heating mechanism will essentially be different and the SAR may decline instead [13,14]. The size of our nanoparticles is about 10 nm, which means that thermogenesis should obey the Brownian relaxation mechanism. However, once the nanoparticles aggregate, the mechanism will become the Neel mechanism so that the thermogenesis is enhanced. Thia is the reason that the heating performance of our sample in the acidic condition (pH = 3) without the BSA adsorption is better than that with the BSA adsorption. Under the basic condition (pH = 8), the BSA adsorption weakened the aggregation of magnetic nanoparticles. Because the size of colloidal aggregates is obviously larger than the critical size, the effect of BSA adsorption is to increase the thermogenesis of the magnetic nanoparticles under the alternating magnetic field. This finding is obviously valuable for the clinical application of nanoparticle-based hyperthermia and deserves attention from physiatrists.

In the actual hyperthermia application, the magnetic nanoparticles are delivered to the body intravenously, which means the nanoparticles may be automatically coated with the so-called protein corona. In fact, the mechanism of protein corona formation is essentially identical with that of our finding in this article. They both result from the non-specific adsorption of biomolecules onto the surface of nanoparticles. However, the blood constituent is too complex. It contains multiple cells, thousands of proteins, and other matter. Thus, the protein corona can be formed by means of the diverse interactions. Our group previously studied the effect of proteins in the neutral cell culture medium on the surface property of iron oxide nanoparticles [9,10]. The results exhibited that the surface of nanoparticles can adsorb the proteins so that the colloidal stability is enhanced and the surface properties of different nanoparticles reach unanimity, such as ζ potential and hydrodynamic size. These surface properties are vital to the field-mediated assembly. Here, we only used albumin, which shares the largest content in serum to exhibit the influence of protein corona on the field-mediated assembly of magnetic nanoparticles. As is seen from our results, the effect of the protein corona will be stronger than the influence on the field-mediated assembly of nanoparticles in the actual application because the real blood contains many more proteins and other components than the BSA solution. In fact, we also used the whole calf serum to inhibit the assembly of magnetic nanoparticles in the presence of the alternating magnetic field. The results exhibited that the serum has the stronger inhibition effect, bringing about the highly dispersive pattern of nanoparticles after the field treatment.

## 3. Experimental Section

The magnetic nanoparticles and BSA were dispersed into the pure water and their concentrations were 0.068 mg/mL and 0.02 mg/mL, respectively. The magnetic nanoparticles in our experiments were γ-Fe_2_O_3_ colloidal particles (The average size was about 10 nm) synthesized by the co-precipitation method without any additional surface modification, following the previous report of our group [11]. Due to the hydroxylated surfaces, the nanoparticles were stabilized electrostatically. The assembly process was identical with that reported in [1,2]. In the experiments, the volume of colloidal solution for assembly was about 10 μL. The vector of the magnetic field was vertical to the assembly plane, and the samples were characterized after the solvent evaporation. The optical microscope (CCD camera) was used to observe the morphology of the assemblies. The size of assemblies was in microscale, which was suitable to be characterized by the optical method.

The experimental setup was same as that in our previous reports [1,2]. Our alternating magnetic field was generated by a 3-turns coil without magnetic core which was energized very high current. The diameter of coil is 3.5 cm and the silicon wafer supporting the colloidal droplet is about 4 mm × 4 mm. In experiments, the silicon wafer was strictly placed in the middle of the coil. The approximate simulation of field distribution was shown in [2]. Based on the simulation, and that in this case the size of the silicon wafer is much smaller than the coil, the field subject to the silicon wafer in the middle area can be considered uniform. The field intensity was indicated by the excitation current. 200 A excitation current equals to 70 KA/m. However, the data of the excitation current was provided by the manufacturer while the data of field intensity was calculated based on the electromagnetic induction rule. Because equipment able to directly measure the magnetic field intensity at such a high frequency is unavailable, we used here the excitation current to indicate the field intensity. In fact, if the size of the coil and the excitation current are determined, the field intensity is also determined.

The IR spectrum characterization was as follows: 6 mL γ-Fe_2_O_3_ colloidal suspension and 3 mL BSA solution were mixed. Then the pH value was adjusted to 8 or 3 with 0.1 M NaOH or 0.1 M HCl. After centrifugal separation, the sediment was re-dispersed into pure water, and the pH value was re-adjusted to 8 or 3 by 0.1 M NaOH or 0.1 M HCl again. By repeating this process several times, the sediment was finally dispersed into the pure water and the sample was sent for IR characterization.

The measurements of heating property were carried out with the glass fiber thermometer. The heating curve can be recorded automatically by the computer. The glass fiber was dipped into the 5 mL cuvette and the cuvette was placed in the alternating magnetic field.

## 4. Conclusions

In summary, we demonstrate that the adsorption of BSA (bovine serum albumin) on the surfaces of magnetic nanoparticles can effectively make the fibrous aggregation of bare γ-Fe_2_O_3_ nanoparticles turn into the more isotropic aggregation in the presence of an alternating magnetic field by simply mixing the protein and colloidal suspension, which is important in some biomedical applications based on the behavior of magnetic nanoparticles with the electromagnetic field involved. We found the collective calorific behavior of magnetic nanoparticles is relative to the colloidal aggregation state. We believe our preliminary works will enrich the technological application of nanomaterials and provide further understanding on the assembly process in fundamental research. Also, more attention should be paid to the case that protein adsorption on the surface can change the colloidal behavior.

## Supplementary Information
