# Supplementary material for: Exploiting BSA to Inhibit the Fibrous Aggregation of Magnetic Nanoparticles under an Alternating Magnetic Field
Source: Int J Mol Sci. 2013 Mar 12;14(3):5775–83. doi: 10.3390/ijms14035775 (PMC3634418; doi:10.3390/ijms14035775)
Supplement: Supplementary file 1 [file ijms-14-05775-s001.pdf]

Supporting Information

Figure S1. (**a**) is the SEM image of γ-Fe_2O_3 nanoparticle aggregates without BSA addition in the presence of a time-varied magnetic field; (**b**) is the SEM image of γ-Fe_2O_3 nanoparticles aggregates after BSA addition in the presence of time-varied magnetic field. The excitation current of magnetic field generator is 200 A.

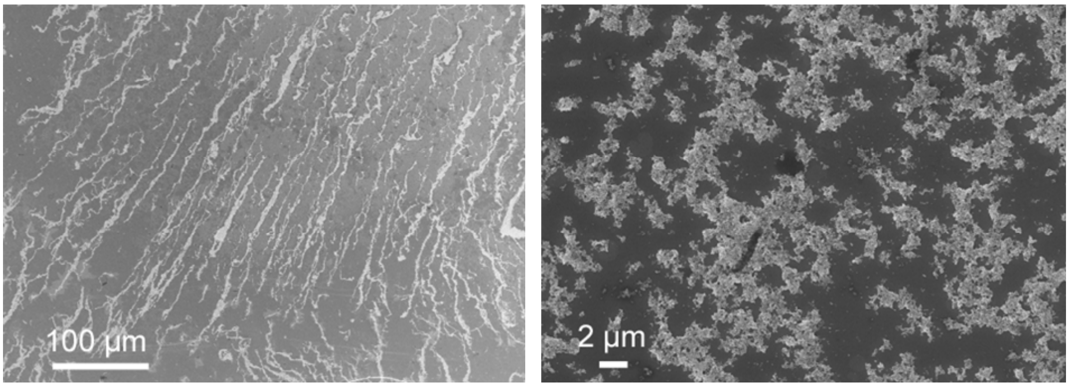

Figure S2. The optical images of γ-Fe2O3 nanoparticle aggregates after BSA addition under different field strengths. (**a**) to (**d**) represent the 200 A, 300 A, 400 A and 500 A excitation current of a magnetic field generator, respectively.

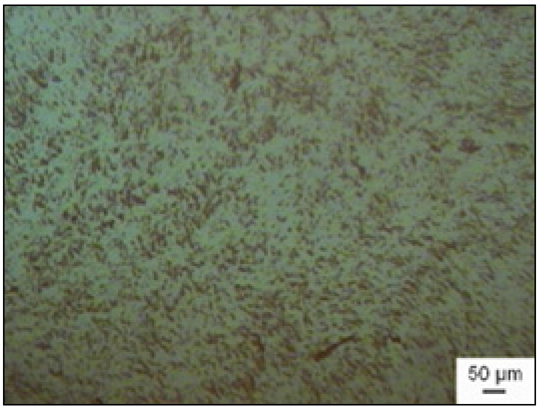

(**a**)

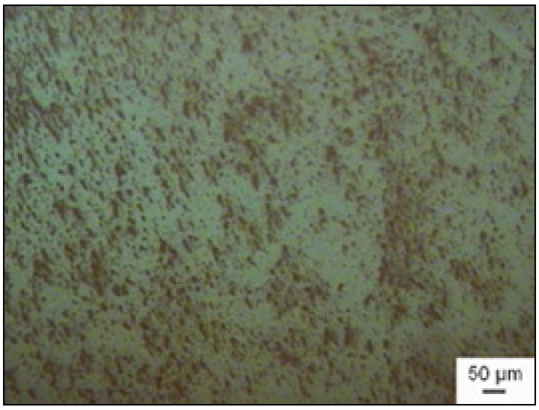

(**b**)

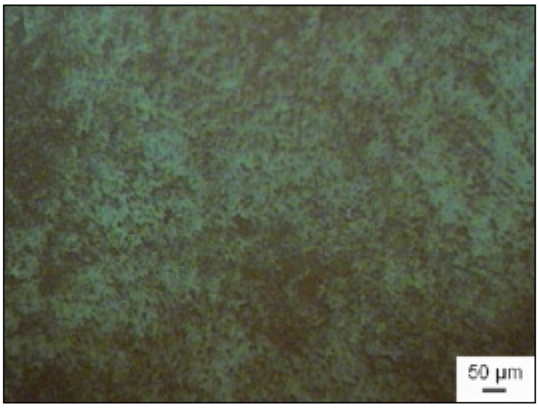

(**c**)

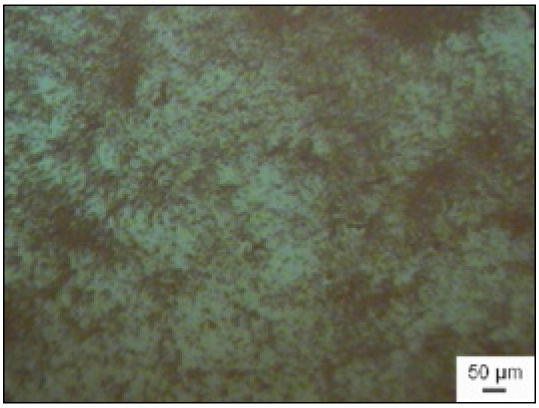

(**d**)

Figure S3. The optical images of γ-Fe_2O_3 nanoparticle aggregates with different pH values after BSA addition. The excitation current of magnetic field generator is fixed in 200 A. The pH values of (**a**)–(**l**) are 6, 4.5, 4, 3.5, 3, 2.5, 7.5, 8, 8.5, 9.5, 10, and 11, respectively.

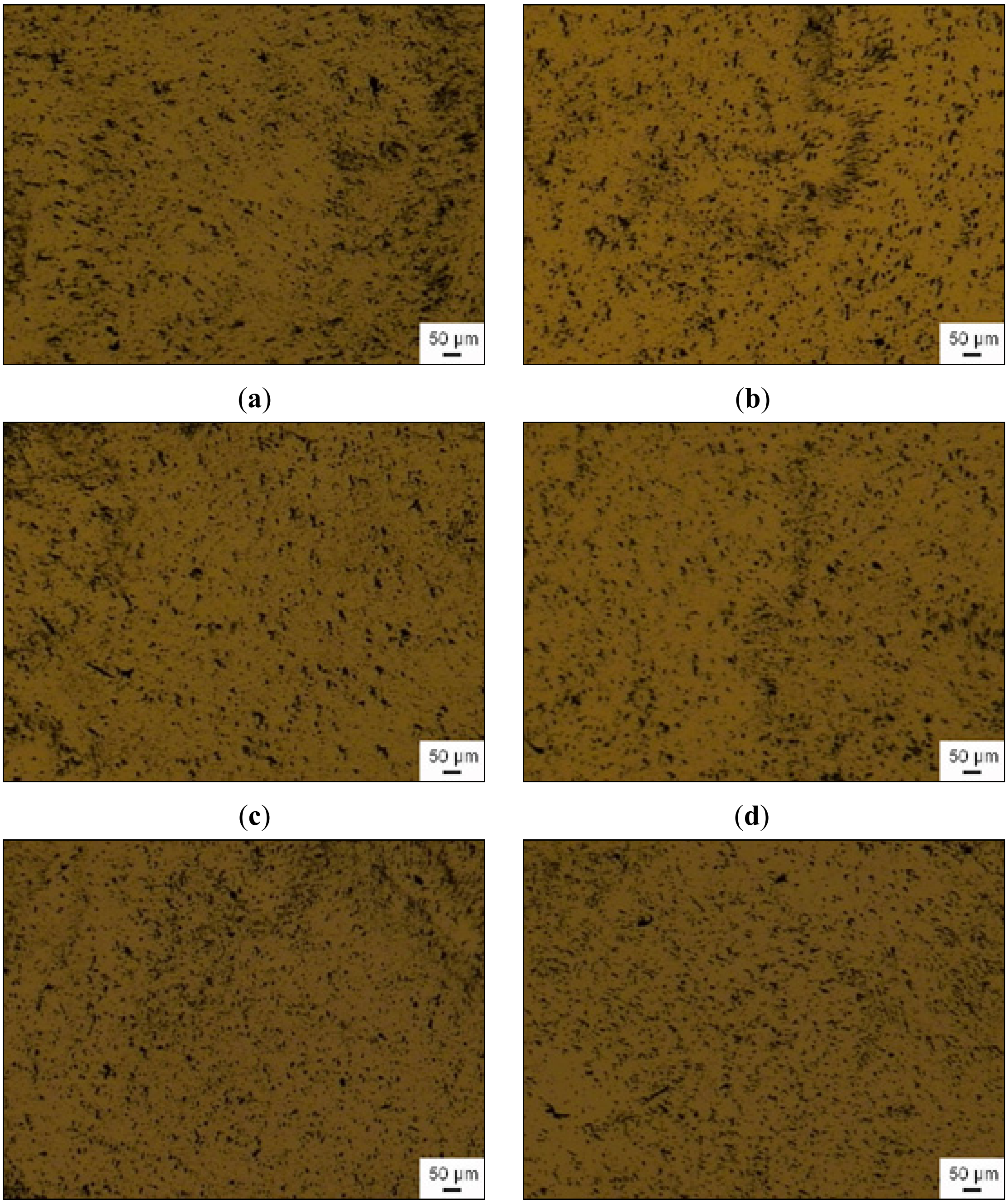

(**a**) (**b**)

(**c**) (**d**)

(**e**) (**f**)

Figure S3. *Cont.*

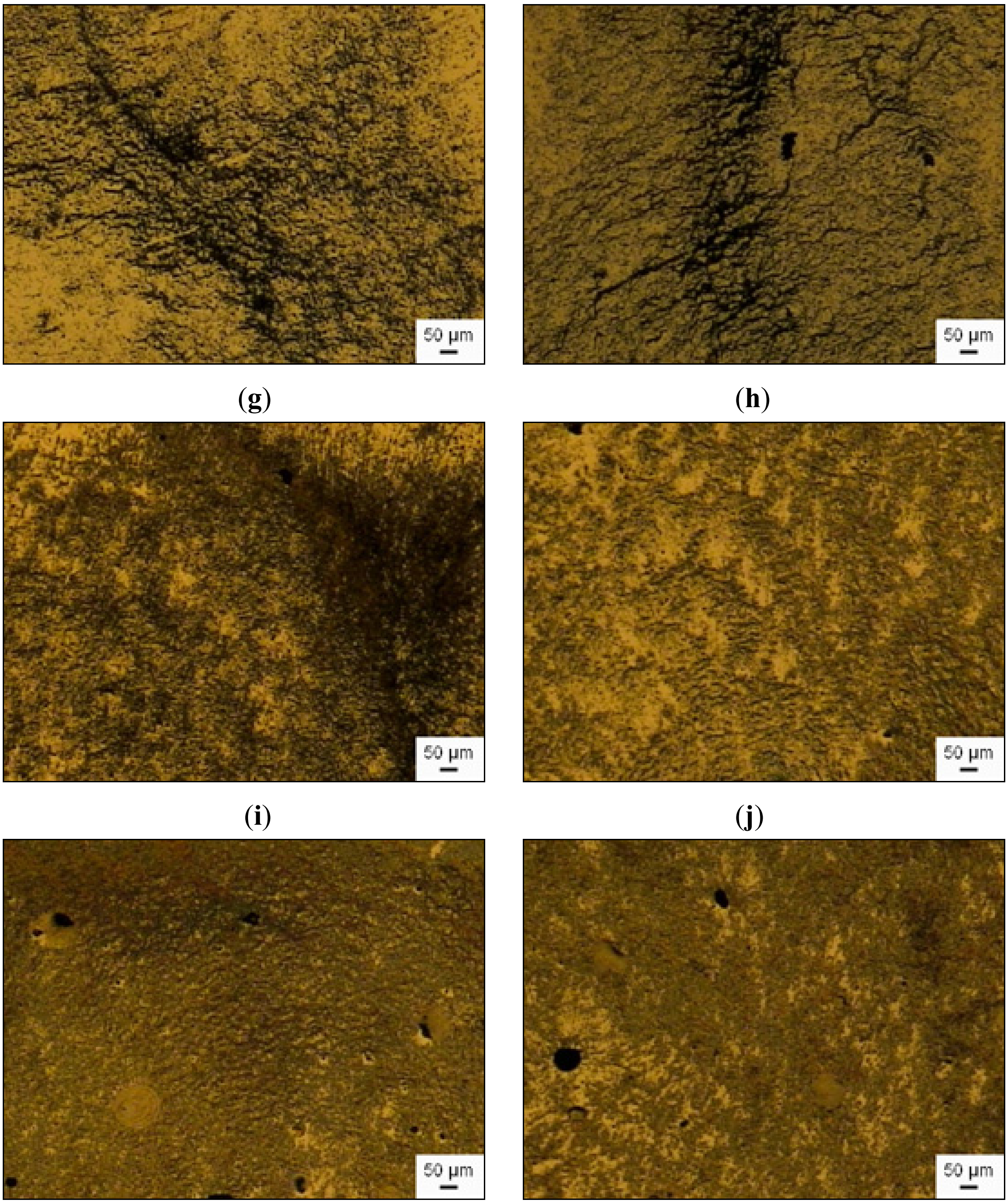

(g) **(h)** **(i)** **(j)** **(k)** **(l)**

© 2013 by the authors; licensee MDPI, Basel, Switzerland. This article is an open access article distributed under the terms and conditions of the Creative Commons Attribution license (http://creativecommons.org/licenses/by/3.0/).
